# Loss of function *JAK1* mutations occur at high frequency in cancers with microsatellite instability and are suggestive of immune evasion

**DOI:** 10.1371/journal.pone.0176181

**Published:** 2017-11-09

**Authors:** Lee A. Albacker, Jeremy Wu, Peter Smith, Markus Warmuth, Philip J. Stephens, Ping Zhu, Lihua Yu, Juliann Chmielecki

**Affiliations:** 1 Foundation Medicine Inc., Cambridge, Massachusetts, United States of America; 2 H3 Biomedicine, Cambridge, Massachusetts, United States of America; Universidade do Minho, PORTUGAL

## Abstract

Immune evasion is a well-recognized hallmark of cancer and recent studies with immunotherapy agents have suggested that tumors with increased numbers of neoantigens elicit greater immune responses. We hypothesized that the immune system presents a common selective pressure on high mutation burden tumors and therefore immune evasion mutations would be enriched in high mutation burden tumors. The JAK family of kinases is required for the signaling of a host of immune modulators in tumor, stromal, and immune cells. Therefore, we analyzed alterations in this family for the hypothesized signature of an immune evasion mutation. Here, we searched a database of 61,704 unique solid tumors for alterations in the JAK family kinases (*JAK1/2/3*, *TYK2*). We used The Cancer Genome Atlas and Cancer Cell Line Encyclopedia data to confirm and extend our findings by analyzing gene expression patterns. Recurrent frameshift mutations in *JAK1* were associated with high mutation burden and microsatellite instability. These mutations occurred in multiple tumor types including endometrial, colorectal, stomach, and prostate carcinomas. Analyzing gene expression signatures in endometrial and stomach adenocarcinomas revealed that tumors with a *JAK1* frameshift exhibited reduced expression of interferon response signatures and multiple anti-tumor immune signatures. Importantly, endometrial cancer cell lines exhibited similar gene expression changes that were expected to be tumor cell intrinsic (e.g. interferon response) but not those expected to be tumor cell extrinsic (e.g. NK cells). From these data, we derive two primary conclusions: 1) *JAK1* frameshifts are loss of function alterations that represent a potential pan-cancer adaptation to immune responses against tumors with microsatellite instability; 2) The mechanism by which *JAK1* loss of function contributes to tumor immune evasion is likely associated with loss of the *JAK1*-mediated interferon response.

## Introduction

Immune evasion is one of the hallmarks of cancer because tumors subvert and even use immune responses to aid growth and invasion [1]. The immune system serves an important role in cancer surveillance and exerts considerable selective pressure on cancers that do develop. Immunosuppressed transplant patients have an increased rate of developing non-viral cancers [2] and the presence of tumor-infiltrating lymphocytes is a positive prognostic factor in several cancer types [3–5]. The presence of infiltrating lymphocytes has been further refined with two important observations. First, higher numbers of effector T cells tends to indicate a positive prognosis, suggesting that anti-tumor responses are antigen specific, while regulatory T cells indicate a negative prognosis [6–8]. Studies in mice have shown that T and B cell deficient animals have a higher incidence of tumors in a chemically induced model; furthermore, these tumors rarely engraft into wild-type hosts although wild-type to wild-type tumor transplants are successful, which further supports the antigen specificity of anti-tumor immune responses [9]. Second, tumors with a high-mutation phenotype resulting from mutation of *POLD1/E* or microsatellite instability (MSI) have both high numbers of neo-antigens and high numbers of tumor infiltrating lymphocytes, suggesting that tumors with more antigens elicit stronger immune responses [10, 11]. Thus, immune evasion and immune editing are important parts of tumor growth and progression, especially for hyper-mutant tumors. One mechanism of immune evasion, checkpoint induction, has been targeted therapeutically with long term responses in a subset of patients [12]. Immune checkpoint inhibition has been successful in cancers with high levels of microsatellite instability (MSI-H) and is approved in diseases such as melanoma, non-small cell lung carcinoma, and metastatic bladder cancer that frequently have high tumor mutational burden [13–15]. Other cell intrinsic mechanisms of immune evasion include loss of proteins involved in antigen presentation, resistance to cell death, and receptor shedding of immune activating receptors [1, 16, 17]. Cell extrinsic mechanisms of immune evasion include secretion of immunosuppressive cytokines or recruitment of immunosuppressive cells such as regulatory T cells or myeloid derived suppressor cells [8, 18, 19]. In addition to subverting the immune response during tumor development, these mechanisms will also likely impact the success of checkpoint inhibitor therapy.

The JAK-STAT family of proteins mediates signaling of the interferon (IFN), IL-6, and IL-2 families of cytokines [20]. JAK-STAT proteins are expressed by immune, stromal, and tumor cells, making them an important transducer of immune signals in nearly all cell types. JAK kinases are constitutively associated with cytokine receptors; cytokine binding results in activation of JAK kinases and phosphorylation of the receptor [21]. Phosphorylated receptors recruit STAT proteins, which are then themselves phosphorylated by the activated JAK kinases. Phosphorylated STAT proteins form homo- and hetero-dimers that translocate into the nucleus to function as transcription factors that regulate gene expression. Because the four JAK kinases (*JAK1*, *JAK2*, *JAK3*, and *TYK2*) mediate signaling for over 20 cytokines, genomic changes that alter their activity can have diverse effects [21]. For example, *Jak1*-deficient mice exhibit perinatal lethality and phenotypes as diverse as defective lymphopoeisis, lack of IFN response, and failure of cardiomyocytes and neuronal cells to respond to growth factors [22]. An immunodeficient patient with early-onset bladder cancer was found to have two different germline biallelic *JAK1* hypomorph alterations [23]. In hematopoietic cancers, the activating mutation *JAK2* V617F drives myeoloproliferative neoplasms and the effects of the JAK-STAT family are considered to be pro-tumorigenic [24]. In solid tumors, activation of STAT3 or STAT5 by mutation or cytokine signaling is pro-tumorigenic, while activation of STAT1 upregulates antigen presentation and has anti-tumorigenic effects [25]. However, the role of the JAK-STAT pathway in solid tumors has been ambiguous due to the low incidence of recurrent mutations in this pathway.

We hypothesized that immune surveillance presents a common selective pressure on high mutational burden tumors and, therefore, tumor-intrinsic immune evasion mechanisms would be enriched in tumors with high mutational burden. We investigated mutations within JAK family kinases suggestive of immune modulation in a collection of 61,704 unique solid tumors. Recurrent *JAK1* frameshift mutations occurred at the highest frequency in endometrial cancers, but were also found in prostate, urinary, gastric, and colon cancers. *JAK1* frameshifts occurred in MSI-H cancers with high mutational burdens. These mutations were observed in over a third of MSI-H cancers, which is in contrast to the low mutation rate of JAK-STAT family members in all solid tumors. *JAK1* is a critical signaling kinase for the interferon, IL-6, and IL-2 pathways. Using The Cancer Genome Atlas (TCGA) data of MSI-H endometrial tumors, we found reduced expression of gene sets associated with interferon gamma, interferon alpha, IL-6, and IL-2 responses in *JAK1* frameshift mutants compared to *JAK1* wild-type tumors. Stomach adenocarcinomas with a *JAK1* frameshift exhibited reductions in both interferon responses but reductions in IL-6 and IL-2 responses were not significant. Both endometrial and stomach tumors with *JAK1* frameshifts also exhibited reductions in gene sets associated with immune surveillance such as the inflammatory response and antigen presentation. Thus, *JAK1* frameshift mutations are a potential pan-cancer adaptation to the immune response that occurs against highly mutated MSI-H tumors. These alterations likely allow tumors to evade the immune response via a lack of antigen presentation and resistance to interferons.

## Methods

### Dataset collection and verification

The Foundation Medicine (FMI) dataset was a set of clinical samples sent to a CLIA (Clinical Laboratory Improvement Amendments)-certified, College of American Pathology (CAP)-accredited, and New York State (NYS)-accredited laboratory (Foundation Medicine, Cambridge, MA) for next-generation sequencing [26]. Samples were subjected to an independent pathology review to verify the correct diagnosis. DNA was extracted and hybrid capture for all coding exons of 287 or 395 genes plus 19 or 31 introns frequently rearranged in cancer was performed; both versions of the test captured *JAK1/2/3*. Libraries were sequenced to a median depth >500X. Analysis for genomic alterations was performed as previously described [26]. Natural germline variants from the 1000 Genomes Project (dbSNP135) were removed. To maximize mutation-detection accuracy (sensitivity and specificity) in impure clinical specimens (≥20% tumor nuclei), the test was previously optimized and validated to detect base substitutions at a ≥5% mutant allele frequency (MAF) with 99% sensitivity, indels (1-40bps) at ≥10% MAF with 98% sensitivity, focal homozygous deletions and amplifications (≥8 copies) with > 95% sensitivity, and select gene fusions with >99% sensitivity [26, 27]. Germline variants without clinical significance were further filtered by applying an internal algorithm to determine somatic/germline status [28].

### Frameshift identification in The Cancer Genome Atlas data

BAM files of Exomeseq for TCGA (http://cancergenome.nih.gov) cohort of uterine corpus endometrial cancer (UCEC), stomach adenocarcinoma (STAD) and colon adenocarcinoma (COAD) were downloaded from Cancer Genomics Hub (https://cghub.ucsc.edu/). GTFuse from Annai Systems is used to get sliced bam files and retrieve only reads of the interested regions such as *JAK1* gene region in this case. *JAK1* mutations for samples with matched normal were called using VarDict [29].

### TCGA data analysis

Except for *JAK1* frameshifts, we analyzed publically available TCGA data for Clinical (MSI and survival), RNASeqV2, and Somatic Mutations. RNA expression was measured using the “scaled estimate” column. Data sets were downloaded on June 1, 2016.

### Cancer cell line encyclopedia (CCLE) RNAseq data

CCLE RNAseq data is downloaded from Omicsoft Corporation (http://www.omicsoft.com/). The FPKM gene expression value provided by Omicsoft is converted to transcripts per million (TPM) using the following formula [30]:

$${TPM}_{i}=(\frac{{FPKM}_{i}}{\sum_{j}{FPKM}_{j}})∙10^{6}$$

### Gene expression and gene set enrichment analysis (GSEA)

Tumors samples from TCGA or cell lines from the CCLE [31] were divided into *JAK1* frameshift and wild-type groups. Gene expression was compared using Student’s t as the distance metric (difference in mean between the two groups divided by standard deviation). Genes were then rank ordered and analyzed using the GseaPreRank tool in GSEA 2.2.2 [32, 33]. We analyzed gene sets in Hallmarks version 5.1 using the weighted enrichment statistic and a custom set of immune genes using the weighted p2 enrichment statistic since the immune gene sets were small. Immune gene sets are provided (S1 Appendix). Note the MHCI gene set is referred to as antigen presentation in the text.

The IFN Gamma response in Figs 3 and 5 is the sum of z-scores for the top 25 genes ranked by the Student’s t for that disease.

### Statistics and visualization

Statistics were calculated using RStudio 0.99 [34], R 3.2.1 [35]; Scipy version 0.15.1, Anaconda version 2.3.0 [36], in Python 2.7.10; or GraphPad Prism. Data were visualized using the cBioportal lollipop plot generator [37, 38], R standard plotting functions and package NMF [39] to display heatmaps, or Matplotlib version 1.4.3 [40] in Python. Not all samples in TCGA have all of the required information (i.e. MSI, RNA-Seq); therefore, TCGA cohorts were adjusted to include the maximum number of samples for a figure panel based on the data required to perform the analysis and the data available for each sample.

### Ethics approval and consent to participate

This study, including the consent procedure, was reviewed and approved by the Western Institutional Review Board (WIRB). For samples within the Foundation Medicine dataset, written patient consent was obtained at the time of testing. Patients were not consented for release of raw sequencing data.

### Availability of data and material

Patients were not consented for release of sequence information by Foundation Medicine. A subset of curated Foundation Medicine mutation data has been released through the National Cancer Institute’s Genomics Data Commons: https://portal.gdc.cancer.gov Program: FM

The results published here are in part based upon data generated by TCGA Research Network: http://cancergenome.nih.gov/ [41].

## Results

### *JAK1* frameshifts are a recurrent alteration in cancers with microsatellite instability

**Fig 1:**
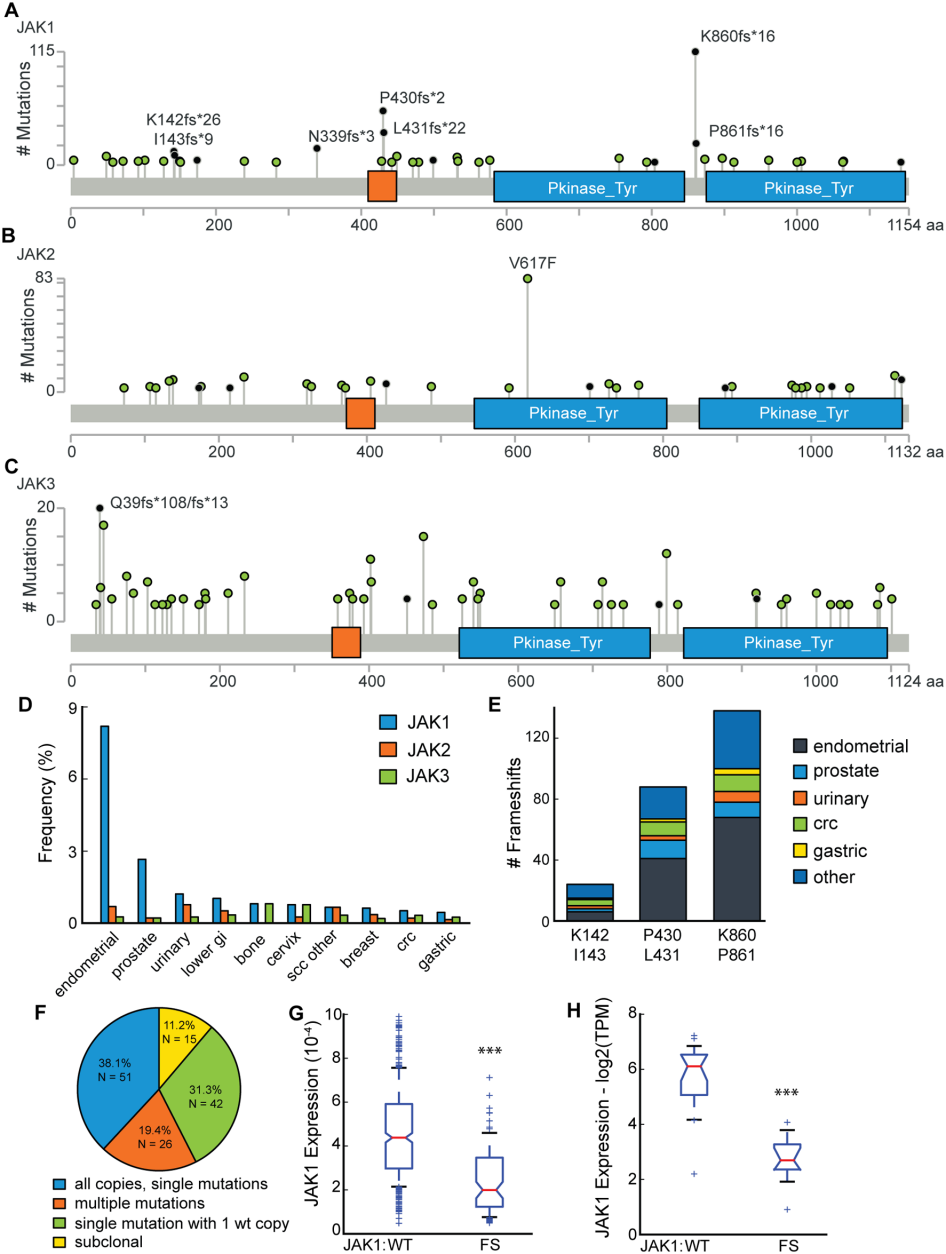
Recurrent *JAK* frameshift alterations in solid tumors. (A-C) Incidence of mutations by amino acid (only positions with >2 tumors mutated shown) in (A) *JAK1*, (B) *JAK2*, and (C) *JAK3*. (D) The frequency of *JAK1/2/3* frameshift mutations in different tumor types, scc other = squamous cell carcinoma of the eye, penis, trachea, vagina, vulva, or unknown primary. (E) Number of *JAK1* frameshifts observed in the three most common hotspots by disease type. (F) Pie chart classifying *JAK1* alterations by the number of *JAK1* copies mutated. (G, H) Box and whiskers plots of *JAK1* expression in endometrial (G) tumors from TCGA, expression measure is scaled estimate and (H) CCLE cell lines, expression measure is log2 of transcripts per million (TPM). The red line defines the median, box defines the quartiles and the whiskers define the 10^th^ and 90^th^ percentiles. The notch in the box provides a 95% confidence interval around the median. (*** = *P* < 10^−4^, Mann-Whitney U test).

We analyzed the spectrum of mutations in *JAK1*, *JAK2*, and *JAK3* across a collection of 61,704 solid tumors that underwent genomic profiling as part of standard clinical care (Foundation Medicine / FMI dataset). Recurrent frameshift mutations occurred in *JAK1* at two hotspots, P430/L431 (n = 90) and K860/P861 (n = 135), and less frequently at K142/I143 (n = 24) and N339 (n = 17) (Fig 1A, only positions with >2 mutations plotted). These hotspot frameshifts occurred at mononucleotide tracts of length 7 (poly T: N339, poly G: P430/L431) and 8 (poly T: K142/I143, K860/P861); the protein annotations spanned two amino acids because frameshift deletions were called as K142, P430, or K860, while frameshift insertions were called as I143, L431, or P861, respectively. Several non-recurrent nonsense mutations in *JAK1* were also observed; however, these usually co-occurred with a P430/L431, K860/P861, or K142/I143 *JAK1* frameshift (data not shown). *JAK2* exhibited the well-described V617F hotspot (Fig 1B). Mutations were rare in *JAK3*; a Q39 frameshift occurred 20 times, with 6 of those in colorectal cancer (CRC) (Fig 1C). Since early termination mutations in *JAK1* were frequent and relatively unstudied, we investigated these alterations further.

Premature terminating mutations (frameshift and nonsense) in *JAK1* were enriched in endometrial cancers (8.2%), prostate cancers (2.7%), urinary cancers (1.2%), and several gastrointestinal tract cancers (gastric, small intestine, and colorectal; <1%, Fig 1D). In contrast, early terminating mutations in *JAK2* and *JAK3* were not disease specific and occurred in fewer than 1% of all disease groups. In total we observed 135 frameshift mutations at the K860/P861 hotspots, with 50.4% (n = 68) occurring in endometrial cancer (Fig 1E). We also observed 90 frameshift mutations at P430/L431, with 45.6% (n = 41) in endometrial cancer. Prostate cancer showed a slightly increased incidence of P430/L431 frameshifts compared to K860/P861 (12 vs. 10, Fig 1E). The P429/P430 proline pair is conserved across JAK family members; we looked for frameshifts at this position and found none in solid tumors (Fig 1A–1C red boxes). A smaller analysis of 3,666 tumors for alterations in *TYK2* identified only two frameshifts in this gene (data not shown). Thus, frameshifts in the JAK family appeared to be JAK1 specific and were recurrent across several solid tumor types.

Loss of function mutations such as frameshifts generally occur in tumor suppressor genes, in which a second event is required to inactivate the remaining wild-type allele. Therefore, we analyzed the mutant allele frequency of *JAK1* early terminating mutations and *JAK1* ploidy to determine the cancer cell fraction in tumor samples. This analysis was limited to samples with greater than 40% tumor purity to increase accuracy. Of samples with a mutation resulting in premature termination of *JAK1*, 38.1% (51/134) had a homozygous mutation in all copies of *JAK1*, thus inactivating the protein in these samples (Fig 1F). Another 19.4% (26/134) of samples had two truncating mutations in *JAK1* and neither was present in all copies. Unfortunately, we cannot assess whether these mutations are on the same or different alleles. At least one copy of *JAK1* remained functional in 31.3% (42/134) of tumors that had a clonal *JAK1* frameshift event. Finally, 11.2% (15/134) of *JAK1* frameshift samples had a subclonal mutation defined as less than 0.75 copies (Fig 1F). In total, 58% of tumors with *JAK1* alterations likely contained inactivation of both *JAK1* alleles and an additional 31% had lost one copy. To further assess if the *JAK1* frameshift mutations were loss of function events, we evaluated expression of *JAK1* mRNA in endometrial tumors from TCGA and endometrial cell lines from the CCLE. *JAK1* mRNA expression was significantly downregulated in both endometrial tumor samples and endometrial cell lines with a *JAK1* frameshift compared to *JAK1* wildtype endometrial samples (Fig 1G and 1H, tumor: *P* < 10^−18^, cell line: *P* < 10^−4^, Mann-Whitney U test). We concluded that multiple mutations in *JAK1* and reduced expression were indicative of loss of function mutations commonly found in tumor suppressor genes.

**Fig 2:**
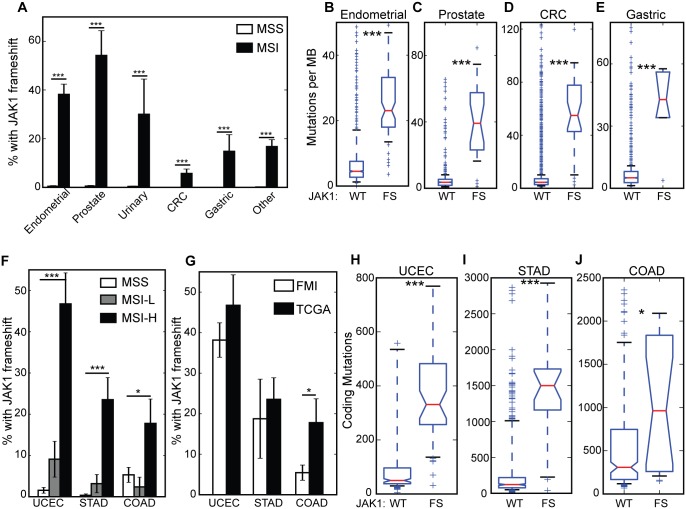
Association of *JAK1* frameshifts with MSI and high tumor mutational burden. (A) Frequency of *JAK1* frameshifts by tumor type stratified by MSS/MSI-H status. Error bars show standard error. (*** = *P* < 10^−4^, Fisher’s exact test) (B-E) Box and whiskers plots of mutations per Mb in (B) endometrial, (C) prostate, (D) CRC, and (E) gastric cancers. The red line defines the median, box defines the quartiles and the whiskers define the 10^th^ and 90^th^ percentiles. The notch in the box provides a 95% confidence interval around the median (*** = *P* < 10^−4^, Mann-Whitney U test). (F) Frequency of *JAK1* frameshifts by tumor type stratified by MSS/MSI-L/MSI-H status (*** = *P* < 10^−4^, * = *P* < 0.05, Fisher’s exact test). (G) Frequency of *JAK1* frameshifts in MSI-H tumors from the FMI and TCGA cohorts (* = *P* < 0.05, Fisher’s exact test). (H-J) Box and whiskers plots of coding mutations per exome in (H) endometrial, (I) stomach, and (J) colon adenocarcinomas (* = *P* < 0.05, *** = *P* < 10^−4^, Mann-Whitney U test).

Deficiencies in the mismatch repair pathway lead to frequent frameshift mutations particularly at mono and di-nucleotide microsatellite repeats, otherwise known as microsatellite instability (MSI). Since frameshifts in *JAK1* occurred at 7-mer and 8-mer mononucleotide repeats, we tested the association between MSI status and the presence of a *JAK1* frameshift alteration in a subset of samples for which MSI status was available. Diagnostically, MSI is scored as microsatellite stable (MSS), MSI-low (MSI-L), or MSI-high (MSI-H); however, for our initial analysis we excluded MSI-L samples. *JAK1* frameshifts were significantly enriched in MSI-H endometrial cancers (38%; 50/131) versus microsatellite stable (MSS) endometrial cancers (<1%; 3/657), (Fig 2A, *P* = 1.78 x 10^−39^, Fisher’s exact test). MSI-H prostate and urinary cancers also showed high rates of *JAK1* frameshifts: 54% (13/24) and 30% (3/10), respectively (Fig 2A). In gastrointestinal cancers, *JAK1* frameshifts were observed in 6% (9/158) and 15% (4/27) of MSI-H colorectal and gastric cancers, respectively (Fig 2A). MSI is rare in other tumor types such as lung cancer; however, 17% (29/173) of other tumors that were MSI-H also had a *JAK1* frameshift. The co-occurrence of *JAK1* frameshifts and MSI-H was statistically significant in all diseases (Fig 2A, *P* < 10^−4^ for all diseases, Fisher’s exact test). MSI-H tumors exhibit increased mutation rates; therefore, we also tested the association of *JAK1* frameshifts with tumor mutational burden (TMB). In endometrial cancers, *JAK1* frameshifts were associated with a significantly higher TMB (median 23.1 mutations per Mb) compared to samples without a JAK1 frameshift (median 4.5 mutations per Mb; Fig 2B, *P* = 5.0 x 10^−39^, Mann-Whitney U test), which is consistent with the increased TMB of MSI-H tumors [42]. Significantly, increased TMB in *JAK1* frameshift samples was also observed in prostate, colorectal, and gastric cancers (Fig 2C–2E, *P* < 0.001, all groups, Mann-Whitney U test). Thus, *JAK1* frameshifts are a likely pan-cancer alteration that occur primarily in MSI-H cancers with high mutational burden.

### Confirmation of *JAK1* frameshifts in TCGA data

We sought to confirm our results in a cohort of tumors from TCGA and independently called *JAK1* frameshifts in these samples (see Methods). FMI disease types were realigned to match TCGA categories. In endometrial adenocarcinoma (UCEC), 3/321 (0.9%) MSS samples, 4/44 (9.1%) MSI-L samples, and 79/169 (46.7%) MSI-H samples had a frameshift alteration in *JAK1*, which was statistically significant when comparing MSS and MSI-H (Fig 2F, *P* < 0.0001, Fisher’s exact test). In stomach adenocarcinoma (STAD), 1/293 (0.3%) MSS samples, 2/63 (3.2%) MSI-L samples, and 20/85 (23.5%) MSI-H samples had a frameshift in *JAK1*, which was also statistically significant when comparing MSS and MSI-H groups (Fig 2F, *P* < 0.0001, Fisher’s exact test). In colon adenocarcinoma (COAD), 8/151 (5.3%) MSS, 1/42 (2.4%) MSI-L, and 8/45 (17.8%) MSI-H samples had a frameshift in *JAK1*, which narrowly met the cutoff for statistical significance due to the large number of *JAK1* alterations in MSS COAD samples (Fig 2F, *P* = 0.0126, Fisher’s exact test); in the FMI data set we did not observe any *JAK1* frameshifts in MSS CRC samples. Finally, we could not confirm the association of *JAK1* frameshifts and MSI in prostate cancer since only 3 *JAK1* frameshift mutations were detected out of 498 samples tested. Furthermore, MSI status was not provided for TCGA prostate cancer samples. Comparison of MSI-H tumors between the FMI and TCGA cohorts showed similar incidence of *JAK1* frameshifts in UCEC and STAD, while the incidence of *JAK1* frameshifts in COAD was significantly greater in TCGA (Fig 2G, COAD *P* = 0.0144, Fisher’s exact test). We also analyzed the association between TMB and *JAK1* frameshift alterations. In the TCGA cohort, we defined coding mutations as non-silent, non-splice site mutations across the genome to be consistent with our measure of TMB in the FMI cohort. In UCEC, TMB was significantly higher in samples with a *JAK1* frameshift (Fig 2H; *P* = 1.8 x 10^−6^, Mann-Whitney U test). STAD, and to a lesser extent COAD, also exhibited higher TMBs in samples with a *JAK1* frameshift (Fig 2I and 2J; STAD: *P* = 2.0 x 10^−9^, COAD: *P* = 0.02). Thus, we confirmed with an independent cohort from TCGA that *JAK1* frameshifts are found primarily in MSI-H tumors that have a high TMB with a similar incidence to the FMI cohort.

### Endometrial tumors with *JAK1* frameshifts exhibit decreased expression of IFN response and immune cell associated genes

To study the functional effects of *JAK1* frameshifts on tumor phenotype, we compared mRNA expression profiles of *JAK1* frameshift and *JAK1* wild-type samples from MSI-H UCEC samples in the TCGA dataset. We used gene set enrichment analysis (GSEA) to analyze pathways that were altered in samples with *JAK1* frameshift mutations. Gene expression changes were rank ordered using Student’s t as the signal to noise metric (S2 Appendix, tab UCEC). In UCEC, *IRF9* was the most significantly downregulated gene in samples with *JAK1* frameshifts; this protein forms a complex with STAT1 and STAT2 to mediate IFN induced transcription. *JAK1* was the 7^th^ most significantly down regulated gene. In the Hallmark collection of gene sets, ten gene sets were significantly decreased in *JAK1* frameshift samples (Table 1, *P* < 0.05 after family wise error rate (FWER) correction). All GSEA results are shown in an additional file (S3 Appendix). *JAK1* mediates signaling in four of these gene sets including IFNγ, IFNα, IL-6, and IL-2 pathways (Table 1). Five of these gene sets were related to immune responses including inflammatory response, *TNFA* signaling, allograft rejection, complement, and coagulation (Table 1). Four gene sets were significantly increased in the *JAK1* frameshift samples that were related to growth and division including E2F targets, G2M checkpoint, fatty acid metabolism, and mitotic spindle (Table 1). Since five significantly altered gene sets were related to immune responses, we developed a custom set of gene signatures for GSEA derived from a published set of differentially expressed genes in immune cells [43] and manually curated an antigen presentation by MHC class I specific gene set (referred to as antigen presentation). These gene sets were small (range 16–61 genes) and GSEA was run with a high penalty. As a result, enrichment at the front of the rank list had to be higher relative to the hallmark gene sets in order to achieve significance. In this analysis, seven immune gene sets showed significantly decreased expression in *JAK1* frameshift samples while none showed significantly increased expression (Table 1). Several of the reduced gene sets have been associated with anti-tumor immunity including antigen presentation, neutrophils, M1 macrophages, and activated NK cells (Table 1). Thus, in MSI-H UCEC, frameshift mutations in *JAK1* resulted in reduced IFN and IL-6 responses as well as reduced M1 macrophages and activated NK cell signatures.

**Table 1:** GSEA of *JAK1* frameshift^+^ and wild-type UCEC.

Analysis	Direction	Gene Set	FWER *P*
Hallmark	Decreased in *JAK1* frameshift	INTERFERON GAMMA RESPONSE	0.000
		INTERFERON ALPHA RESPONSE	0.000
		IL6 JAK STAT3 SIGNALING	0.000
		INFLAMMATORY RESPONSE	0.000
		ALLOGRAFT REJECTION	0.000
		TNFA SIGNALING VIA NFKB	0.000
		COMPLEMENT	0.000
		IL2 STAT5 SIGNALING	0.003
		KRAS SIGNALING UP	0.007
		COAGULATION	0.016
	Increased in *JAK1* frameshift	E2F TARGETS	0.000
		G2M CHECKPOINT	0.000
		MITOTIC SPINDLE	0.045
		FATTY ACID METABOLISM	0.047
Immune	Decreased in *JAK1* frameshift	ANTIGEN PRESENTATION	0.000
		NEUTROPHILS	0.000
		PLASMA CELLS	0.000
		MONOCYTES	0.003
		MACROPHAGES M1	0.005
		NK CELLS ACTIVATED	0.006
		DENDRITIC CELLS ACTIVATED	0.033
		B CELLS MEMORY	0.033

GSEA results specifying gene set and the direction of enrichment. Family wise error rate (FWER) *P* values below 0.001 are represented as 0.000.

**Fig 3:**
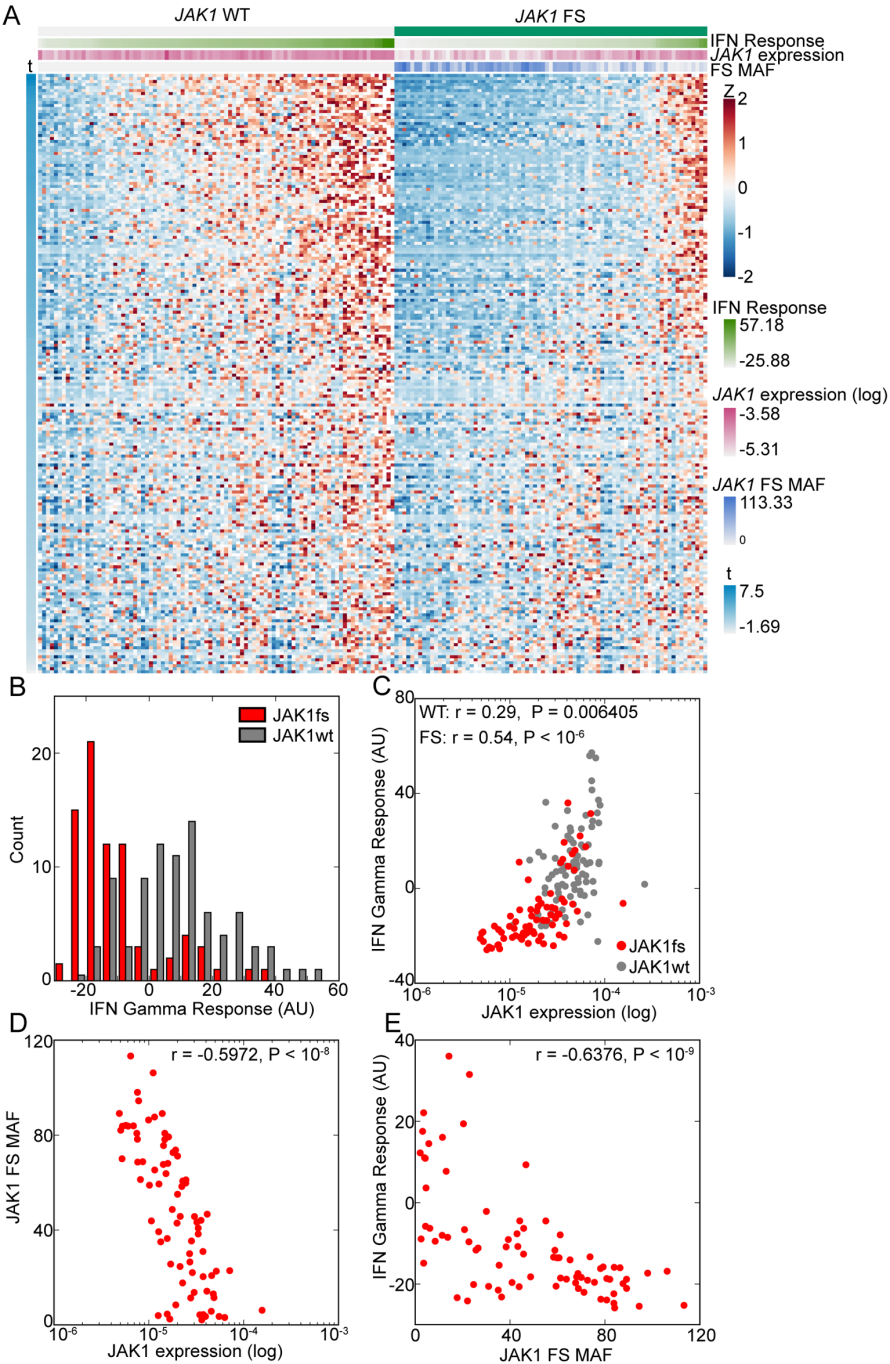
Sample level gene expression changes in MSI-H UCEC samples with a *JAK1* frameshift. (A) Heatmap of MSI-H, UCEC tumors (columns). Marker rows: 1) Samples with a *JAK1* frameshift are denoted in green. 2) IFN Response, after *JAK1* frameshift samples are ordered by IFN Response. 3) Log10 of *JAK1* expression. 4) *JAK1* frameshift (FS) MAF. Gene expression was scaled by Z scoring and the order of genes (rows) is determined by the signal to noise metric for GSEA analysis (t). Negative t indicated decreased expression in *JAK1* frameshift samples. Only genes in the HALLMARK IFN GAMMA RESPONSE are shown. (B) Histogram of IFN Gamma Response in arbitrary units (AU). (C) Scatter plot of samples for *JAK1* expression and IFN Gamma Response. Pearson’s r and associated P values are shown. (D) Scatter plot of samples for *JAK1* expression and *JAK1* FS MAF. Pearson’s r and associated P values are shown. (E) Scatter plot of *JAK1* frameshift samples for *JAK1* FS MAF and IFN Gamma Response. Pearson’s r and associated P value is shown.

Since GSEA measures average expression, we also examined the expression of mRNAs in the IFNγ response gene set for individual samples. This plot showed similar down regulation of these genes for most *JAK1* frameshift tumors (Fig 3A, marker column 1: green *JAK1* frameshift positive). However, a subset of samples with *JAK1* frameshifts exhibited an IFNγ response signature (Fig 3A, right). One possible explanation for this observation is that the *JAK1* loss of function alteration in these tumors was subclonal or affected only one copy, which was a frequent occurrence in the FMI dataset (Fig 1F). We quantified the IFNγ response signature in each sample (see Methods) and observed a bimodal distribution for samples with a *JAK1* frameshift (Fig 3B, red bars) but a unimodal distribution for samples without a *JAK1* frameshift (Fig 3B, grey bars). We investigated the correlation of the IFNγ response signature with *JAK1* expression since expression is reduced in cell lines and tumor samples with a *JAK1* frameshift (Fig 1G and 1H). We observed a significant correlation between the expression of *JAK1* and IFNγ response in samples with or without a *JAK1* frameshift (Fig 3C, JAK1 frameshift: Pearson’s r = 0.54, *P* = 2.5 x 10^-7^, red circles; *JAK1* non-frameshift: Pearson’s r = 0.29, *P* = 0.006, gray circles; Fig 3A, marker row 2: IFNγ response, marker row 3: *JAK1* expression). Therefore, we also examined the mutant allele frequency (MAF) of *JAK1* frameshifts. Sample MAF was the sum of MAF’s for all *JAK1* frameshifts in a sample, with low numbers indicative of subclonal or heterozygous mutations and high numbers indicative of homozygous loss of *JAK1*. As expected, *JAK1* MAF was inversely correlated with *JAK1* expression (Fig 3D, Pearson’s r = -0.597, P = 6.26 x 10^−9^). *JAK1* frameshift MAF had a statistically significant inverse correlation with IFNγ response (Fig 3E, Pearson’s r = -0.64, P = 2.63 x 10^−10^, Fig 3A marker row 4: *JAK1* frameshift MAF). From these data, we conclude that homozygous loss of *JAK1* leads to loss of the IFNγ response gene set, consistent with a role for *JAK1* as a tumor suppressor in this context.

We next assessed whether overlap of gene sets with the 200-gene IFNγ response set resulted in false enrichment of similar gene sets. The overlap of the IFNγ response gene set with hallmark gene sets that were altered in both tumors and cell lines was highly variable, with 73/97 genes for IFNα, 37/200 for allograft rejection and inflammatory response, and 5/200 for EMT. To control for overlap with the IFNγ response gene set, we removed overlapping genes and ran the GSEA analysis again. All hallmark gene sets with a FWER *P* < 0.001 in Table 1 remained significant, while the *P* value for KRAS, IL2 signaling, and coagulation increased above 0.05. There was also overlap of the IFNγ response gene set with immune gene sets: 9/16 for antigen presentation, 13/48 for M1 macrophages, 8/59 for activated NK cells, and 3/60 for neutrophils. Analysis of these gene sets after removing genes that overlap with IFNγ response resulted in M1 macrophages and activated NK cells having a FWER *P* > 0.05, and the antigen presentation gene set became too small to test. However, IFNγ is a known activator of NK cells and is involved in M1 macrophage polarization; therefore, the reduction in enrichment for these gene sets was expected and we concluded that overlap with the IFNγ response gene set was not a confounder of these results.

**Fig 4:**
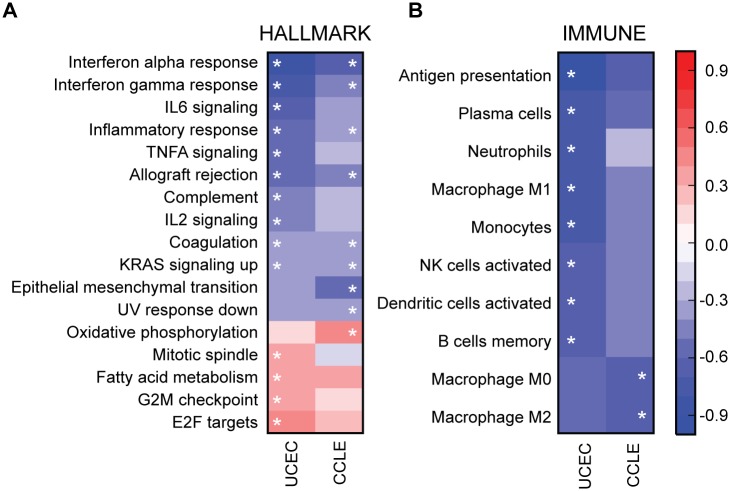
GSEA results in UCEC and CCLE. Heatmap showing the maximum enrichment score (ES) for (A) Hallmark or (B) Immune gene sets in UCEC and CCLE for significantly altered gene sets. Negative ES indicates expression is decreased in *JAK1* frameshift samples. White star indicates gene set is significantly altered in that disease type (FWER *P* < 0.05).

Gene expression analysis was performed on resection specimens composed of tumor, stromal, and immune components. To establish which gene sets were altered in tumor cells versus stromal or immune components, we performed the same GSEA analysis on 29 endometrial cell lines characterized in the CCLE (HEC1A was excluded because of its relation to HEC1B). Ten of these cell lines (34.5%) had a *JAK1* frameshift. Consistent with the TCGA UCEC tumor samples, *JAK1* and *IRF9* were the first and third most significantly downregulated genes, respectively (S2 Appendix, tab CCLE). Eight hallmark gene sets were significantly downregulated in *JAK1* frameshift samples and five of those were reduced in endometrial tumor samples including IFNα, IFNγ, inflammatory response, allograft rejection, and coagulation (Table 2, S3 Appendix). The trend of upregulated versus downregulated gene sets was also consistent between UCEC and CCLE even if significance was not reached (Fig 4). We also analyzed the immune gene signatures in the CCLE. The majority of immune gene signatures were downregulated in *JAK1* frameshift samples from the CCLE; however, only two gene sets in the CCLE comparison were statistically significant (Fig 4, Table 2, M0 and M2 macrophages). Neither of these gene sets were significantly altered in UCEC tumor samples (Fig 4). Thus, we conclude that changes in the hallmark gene sets were likely reflective of tumor cell intrinsic changes in gene expression while changes in the immune gene sets were likely tumor cell extrinsic.

**Table 2:** GSEA of *JAK1* frameshift^+^ and wild-type CCLE endometrial cell lines.

Analysis	Direction	Gene Set	FWER p-val
Hallmark	Decreased in *JAK1* frameshift	INTERFERON ALPHA RESPONSE	0.000
		EPITHELIAL MESENCHYMAL TRANSITION	0.000
		INTERFERON GAMMA RESPONSE	0.000
		ALLOGRAFT REJECTION	0.000
		KRAS SIGNALING UP	0.000
		INFLAMMATORY RESPONSE	0.001
		COAGULATION	0.013
		UV RESPONSE DN	0.034
	Increased	OXIDATIVE PHOSPHORYLATION	0.000
Immune	Decreased	MACROPHAGES M0	0.008
		MACROPHAGES M2	0.009

GSEA results specifying gene set and the direction of enrichment. Family wise error rate (FWER) *P* values below 0.001 are represented as 0.000.

### Stomach adenocarcinomas with *JAK1* frameshifts exhibit decreased expression of IFN associated genes

**Fig 5:**
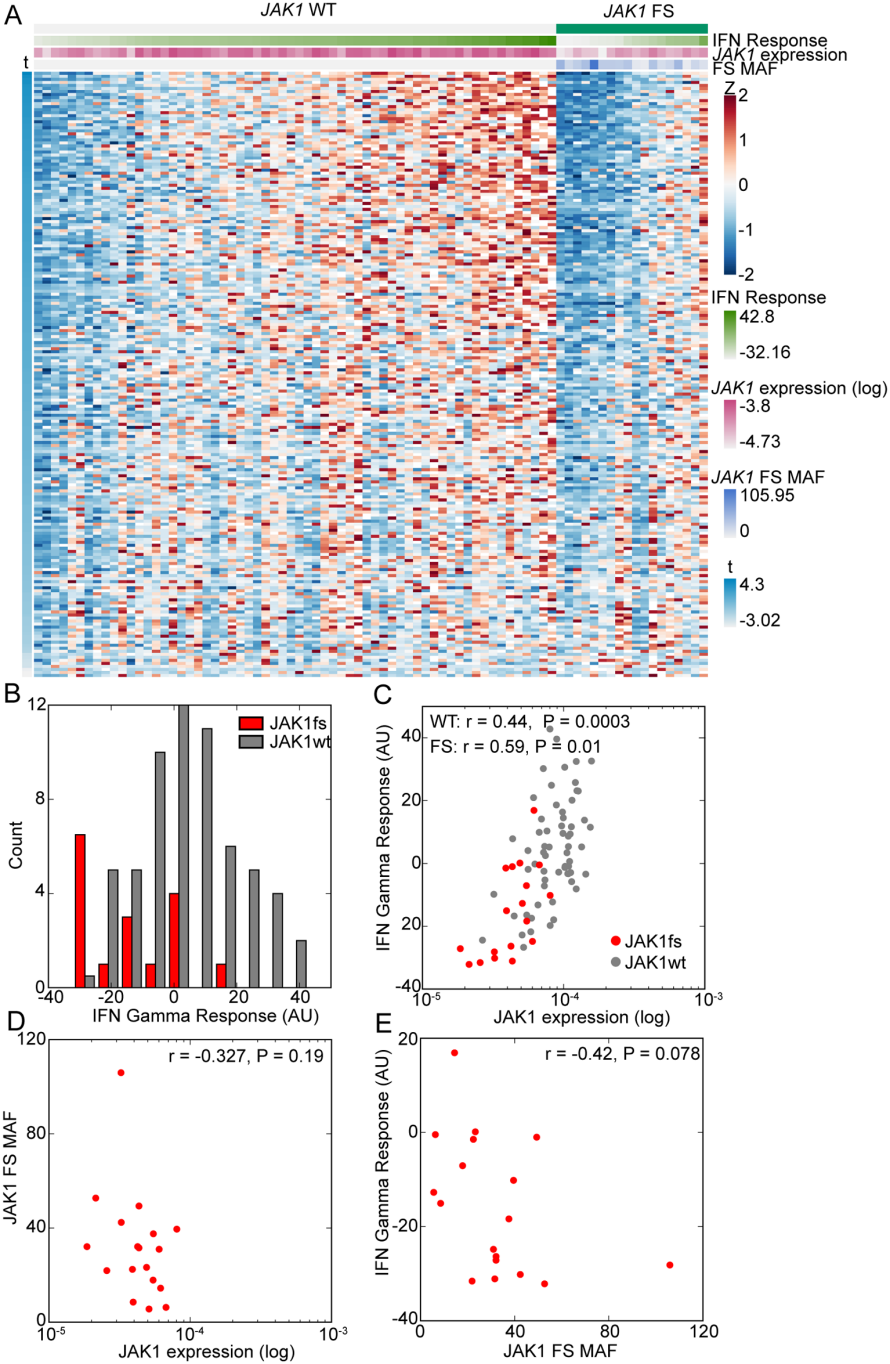
Sample level gene expression changes in MSI-H STAD samples with a *JAK1* frameshift. (A) Heatmap of MSI-H, STAD tumors (columns). Marker rows: 1) Samples with a *JAK1* frameshift are denoted in green. 2) IFN Response, after *JAK1* frameshift samples are ordered by IFN Response. 3) Log10 of *JAK1* expression. 4) *JAK1* frameshift (FS) MAF. Gene expression was scaled by Z scoring and the order of genes (rows) is determined by the signal to noise metric for GSEA analysis (t). Negative t indicated decreased expression in *JAK1* frameshift samples. Only genes in the HALLMARK IFN GAMMA RESPONSE are shown. (B) Histogram of IFN Gamma Response in arbitrary units (AU). (C) Scatter plot of samples for *JAK1* expression and IFN Gamma Response. Pearson’s r and associated P values are shown. (D) Scatter plot of samples for *JAK1* expression and *JAK1* FS MAF. Pearson’s r and associated P values are shown. (E) Scatter plot of *JAK1* frameshift samples for *JAK1* FS MAF and IFN Gamma Response. Pearson’s r and associated P value is shown.

We also analyzed the effect of *JAK1* frameshifts on gene expression in MSI-H STAD. GSEA analysis of MSI-H STAD revealed four significantly down regulated pathways in STAD with a *JAK1* frameshift including IFNα response, IFNγ response, mitotic spindle, and G2M checkpoint (Table 3). Significantly upregulated pathways include the epithelial mesenchymal transition (EMT), cholesterol homeostasis, angiogenesis, and coagulation (Table 3). Analyzing immune signatures showed that antigen presentation and memory B cells were significantly down regulated (Table 3). Gene expression changes were also clear at the sample level with the majority of STAD with a *JAK1* exhibiting reduced expression of genes in the IFNγ response gene set (Fig 5A and 5B). The IFNγ response correlated with *JAK1* expression in samples with and without *JAK1* frameshifts (Fig 5C; *JAK1* frameshift Pearson’s r = 0.59, *P* = 0.01; *JAK1* non-frameshift Pearson’s r = 0.44, *P* = 0.0003). As in UCEC, *JAK1* expression and *JAK1* MAF exhibited an inverse correlation although it did not reach statistical significance (Fig 5D, Pearson’s r = -0.327, *P* = 0.19). The IFNγ response also had a negative correlation with *JAK1* MAF in *JAK1* frameshift samples that trended towards significance (Fig 5E, Pearson’s r = 0.-42, *P* = 0.078). These findings suggest that in STAD, like UCEC, loss of IFN responsiveness requires complete loss of *JAK1*.

**Table 3:** GSEA of MSI WT vs. JAK1 frameshift STAD.

Analysis	Direction	Gene Set	FWER *P*
Hallmark	Decreased in *JAK1* frameshift	INTERFERON ALPHA RESPONSE	0.000
		INTERFERON GAMMA RESPONSE	0.000
		MITOTIC SPINDLE	0.001
		G2M CHECKPOINT	0.038
	Increased in *JAK1* frameshift	EPITHELIAL MESENCHYMAL TRANSITION	0.000
		CHOLESTEROL HOMEOSTASIS	0.000
		ANGIOGENESIS	0.015
		COAGULATION	0.039
Immune	Decreased in *JAK1*	ANTIGEN PRESENTAITON	0.002
		B CELLS MEMORY	0.03

GSEA results specifying gene set and the direction of enrichment. Family wise error rate (FWER) *P* values below 0.001 are represented as 0.000.

**Fig 6:**
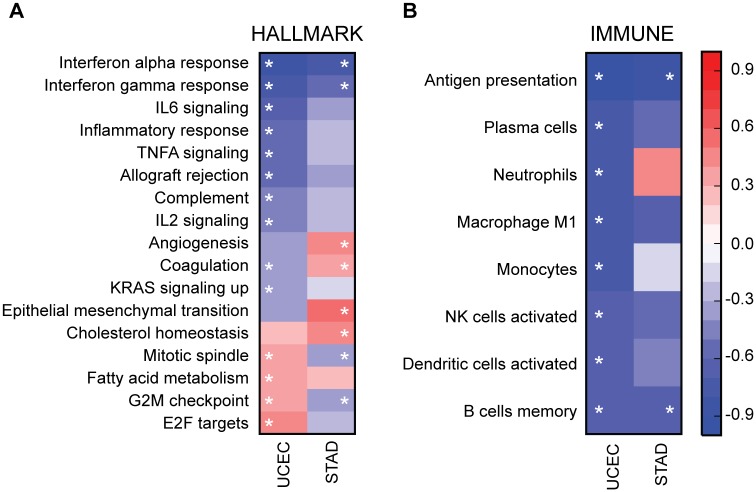
Comparison of GSEA results for UCEC and STAD. Heatmap showing the maximum enrichment score (ES) for (A) Hallmark or (B) Immune gene sets in UCEC and STAD for significantly altered gene sets. Negative ES indicates expression is decreased in *JAK1* frameshift samples. White star indicates gene set is significantly altered in that disease type (FWER *P* < 0.05).

To begin to analyze broader effects of *JAK1* frameshifts, we compared GSEA results of STAD and UCEC. The core effect of IFN unresponsiveness shown by decreases in the IFNα response, IFNγ response, and antigen presentation gene sets were conserved between STAD and UCEC (Fig 6). Immune gene sets were also both generally reduced in STAD and UCEC; however, only two reached significance in STAD. Several gene sets exhibited tumor type specificity. Gene sets associated with proliferation including the mitotic spindle, G2M checkpoint, and E2F targets were increased in *JAK1* frameshift UCEC but decreased in *JAK1* frameshift STAD (Fig 6). In contrast, gene sets reminiscent of a recently described immunotherapy resistant signature (IPRES) such as angiogenesis and EMT gene sets were higher in *JAK1* frameshift STAD but lower in *JAK1* frameshift UCEC (Fig 6) [44]. We conclude that *JAK1* frameshifts result in reduced interferon responses in UCEC and STAD; however, subsequent phenotypic alterations such as proliferation and mesenchymal phenotype are likely cell type specific.

## Discussion

In this study, we showed that loss of function frameshift mutations in *JAK1* may have a role in immune evasion. These frameshifts appear in tumors that are MSI-H and have a high TMB, which are therefore likely to elicit immune responses. We observed a similar incidence of these frameshifts in two independent patient cohorts after normalizing by MSI-H status. We additionally showed alterations in gene expression patterns using TCGA and CCLE data that are suggestive of direct effects such as tumor cell-intrinsic reductions in IFN response genes and indirect effects through alterations to inflammation and macrophage polarization. From these data, we derived two primary conclusions: 1) *JAK1* frameshifts are loss of function alterations that represent a potential pan-cancer adaptation to immune responses against MSI tumors. We observed these frameshifts in cancers where MSI is both common (i.e. endometrial cancer) and rare (i.e. prostate cancer). Furthermore, the frameshift mutations themselves are likely a result of mismatch repair deficiency which causes MSI. MSI-H tumors have a high TMB that causes an immune response that can be mitigated by loss of function mutations in *JAK1*. 2) The mechanism by which *JAK1* loss of function allows immune evasion is likely loss of *JAK1*-dependent interferon responses. *JAK1* is required for interferon induced antigen presentation and growth inhibition [21, 22]. Of the three pathways that utilize *JAK1*, only IFN response was significantly down regulated in both endometrial and stomach cancers. Endometrial cancer cell lines with *JAK1* frameshifts have been shown to be resistant to the effects of IFNγ, including a lack of growth arrest and a failure to up regulate antigen processing and presentation [45]. However, some tumor samples with *JAK1* frameshift mutations in the TCGA dataset exhibited high expression of IFNγ response genes. Using *JAK1* expression and MAF, we showed that these samples likely have heterozygous or subclonal *JAK1* frameshift mutations with complete loss of *JAK1* required to abrogate signaling. About 40% of samples in the FMI dataset had a heterozygous deficiency in *JAK1*. Another explanation for IFN signaling in these samples is *JAK1* independent NF-κB pathway signaling, which has been demonstrated for IFNα treatment [46]. However, most of the genes in the IFN response gene sets have a *STAT1* consensus transcription factor binding site (data not shown) [47], while the *JAK1* independent NF-κB pathway would be expected to activate NF-κB responsive genes. In total, we conclude that *JAK1* frameshift mutations are a potential pan-cancer adaptation to anti-tumor immunity and that these alterations likely result in a lack of antigen presentation and resistance to interferons.

Based on this data, we speculate that *JAK1* frameshifts will also affect treatment with checkpoint inhibitors. Current companion diagnostics for checkpoint inhibitors utilize PD-L1 immunohistochemistry to identify patients likely to respond to these agents in some tumor types. Since PD-L1 (*CD274*) is an IFNγ responsive gene, tumors with a *JAK1* frameshift will likely have reduced expression of PD-L1 and may therefore have a negative result on the companion diagnostic assay. In STAD, high expression of *CD274* in MSI-H *JAK1* frameshift STAD was rare compared to MSI-H *JAK1* wild-type STAD (data not shown). Therapeutically, loss of *JAK1* or MHCI pathway genes was shown to be associated with adaptive resistance to single agent pembrolizumab in a recent study of four relapsed melanoma patients [48]. In these patients, heterozygous point mutations in *JAK1* or MHCI pathway components in the primary sample exhibited loss of heterozygosity in the relapsed sample. Loss of *B2M* was also observed previously in melanoma patients treated with *ex vivo* expanded CD8^+^ T cells and IL-2. Using TCGA data, we found that 71% (60/85) of STAD, 80% (36/45) of COAD, and 31% (52/169) of UCEC had a frameshift in the MHCI pathway; however, the occurrence of these mutations was independent of *JAK1* frameshifts and did not affect our analysis. While the response rate to checkpoint inhibitors has been higher in MSI-H cancers compared to MSS cancers of the same disease type, the determinants of response within MSI-H cancers remain to be described [13]. We speculate that tumors with complete loss of function of *JAK1* will exhibit primary resistance to checkpoint inhibitors. Tumors with heterozygous or subclonal frameshifts in *JAK1* will relapse after acquiring another *JAK1* alteration or undergoing loss of heterozygosity at the *JAK1* locus. Additional data, including paired genomics and immunotherapy treatment outcomes, will be needed to validate these conclusions; nevertheless, our analysis strongly suggests loss of function *JAK1* alterations represents a tumor-intrinsic mechanism of immune evasion and possibly renders MSI-H tumors resistant to checkpoint inhibitors.

*JAK1* frameshift alterations have been described mainly in the context of endometrial cancer [49]. The underlying biochemistry of these alterations is well explained, with frameshifts showing a loss of function phenotype for IFNγ signaling [45]. This study was consistent with knockout studies in cell lines and mice that show *JAK1* is a critical mediator of signaling for the IFN, IL-6, and IL-2 families of cytokines [22]. We extended on these studies by analyzing a database of over 60,000 solid tumors to show that these mutations occur across multiple cancer types and are associated with MSI-H and high TMB. Thus, the previous association of these alterations with endometrial cancer is not unexpected since MSI is frequent in endometrial cancer and *JAK1* frameshifts occur in 40–50% of MSI-H endometrial cancers [42]. The rare occurrence of *JAK1* frameshifts in prostate or urinary cancer can be explained in part by the rarity of MSI in these tumor types. This positive association with MSI strengthens the conclusion that *JAK1* frameshifts are likely immune evasion mutations, and may represent a mechanism through which MSI-H tumors can circumvent immune pressures. Previous reports have shown frequent *JAK1* frameshifts in endometrial cancer but none were observed in CRC, another tumor type with an established MSI-H phenotype [49]. We observed a difference in prevalence between these two diseases but did detect CRC samples with *JAK1* frameshifts. Analyzing TCGA and CCLE expression data with GSEA, we confirmed indirectly the underlying biochemistry in human tumors samples by showing that expression of response genes for IFN and IL-6 families of cytokines are reduced in tumor samples and cell lines with *JAK1* frameshifts. Beyond biochemical effects, reductions in gene sets associated with anti-tumor immunity were also observed in human cancers. Thus, these mutations are not limited to endometrial cancer and the direct effects of IFN; they also occur in MSI-high tumors and result in both unresponsiveness to IFN and a blunted tumor immune response.

Gene expression analysis in this study was performed on tumor samples collected by the TCGA consortium and are thus composed of tumor, immune, and stromal cells. While we adapted previous methods designed to deconvolute expression patterns between cell types in these types of specimens [43, 50], the analysis is still being performed on a mixture of cells. Therefore, gene expression changes we attributed to tumor cell intrinsic processes may instead be tumor microenvironment-relevant. To control for this potential confounder, we performed GSEA on endometrial cell lines, without immune or stromal components, from the CCLE. This analysis suggested that the effects of *JAK1* frameshifts on IFN signaling were likely tumor cell intrinsic whereas decreased expression of activated immune cell genes was likely cell extrinsic. Interestingly, the inflammatory response and allograft rejection gene sets were significantly different in both the cell line and tumor analyses. The changes to these gene sets may simply reflect overlap, known or unknown, with IFN response genes. The cell line data also could not control for gene sets associated with growth and division since cell lines divide continuously and are grown in media with external growth factors. In endometrial tumor samples, we observed increased expression of gene sets associated with cell growth and division in tumors with *JAK1* frameshifts and it is tempting to conclude that unresponsiveness to the growth arrest induced by interferon allows these tumors to grow faster than *JAK1* wild-type tumors. However, given that we were analyzing a mixture of cells, it was unclear which cell types were dividing and we thus have refrained from making conclusions about cell growth and division processes. Despite these limitations, we observed the expected phenotype of IFN unresponsiveness in tumors with a loss of function *JAK1* mutation. The immune alterations we observed were centered on the type or activation state of the immune response. In endometrial cancer, we observed differences in antigen presentation, M1 macrophage differentiation, and activated NK cells, but not resting NK cells or other macrophage types. We speculate these changes in expression of UCEC samples to be the result of tumor-cell extrinsic changes of macrophage polarization away from an anti-tumor M1 phenotype to a more neutral M0 or pro-tumor M2 phenotype. These data are consistent with observations by us and others that MSI-H endometrial cancers had lymphocytic infiltrates regardless of *JAK1* alteration status (data not shown and [51]). In addition, STAD exhibited increased expression of gene sets similar to the IPRES immunotherapy resistance signature [44]. Based on these data, we speculate that *JAK1* frameshift alterations and the resultant unresponsiveness to IFN lead to reduced anti-tumor immune responses.

In this study, we found no evidence for prognostic value for *JAK1* frameshifts in TCGA data and the effect of these mutations on outcome will likely require the incorporation of additional clinical variables. A separate study of MSI-H endometrial cancers also showed no prognostic value of *JAK1* frameshift mutations in patients receiving standard clinical care [51]. MSI-H tumors have a better prognosis than MSS tumors in colorectal cancer [16]. In endometrial cancer, MSI-H tumors have an intermediate prognosis, with *POLE* mutated cancers having the best prognosis and serous-like cancers having the worst [42]. Furthermore, mutation of the *B2M* gene in MSI-H colon cancers is an independent positive prognostic factor. *B2M* mutant colon cancers formed large primary tumors but rarely metastasized [16]. Clinically, many MSI-H cancers will likely be cured by surgery and adjuvant chemotherapy. However, a proportion will progress, and in this context, *JAK1* frameshifts may play a role in immune evasion. This may also be true for other gastrointestinal cancers where *JAK1* and other MHCI pathway components may be contributing to this phenotype. *JAK1* frameshifts may also play a role in evasion to checkpoint inhibitors.

## Supporting information

S1 Appendix Gene sets for analysis of immune cell types. This file contains the name of the gene set and the genes contained in the set. This file can be directly imported into the GSEA software and alternately can be viewed with Excel or a Text Editor.
(GMT)

S2 Appendix Gene rank by student’s t for GSEA. This file contains the rankings of genes for GSEA. There are five tabs for the comparisons included in the text including UCEC: JAK1 frameshift +/- MSI endometrial cancers, CCLE: JAK1 frameshift +/- endometrial cell lines from the CCLE, and pairwise comparisons for MSI STAD cancers: MSI-WT vs. MSI-MHCI, MSI-WT vs. MSI-JAK1, and MSI-JAK1 vs. MSI-MHCI.
(XLSX)

S3 Appendix Complete GSEA results. This file contains complete GSEA results from the comparisons described in the paper in 20 tabs. The naming convention for the tabs is as follows: group (described in legend for File 2), gene set tested (Immune or Hallmark), enrichment direction (positive or negative). Positive enrichments are higher in the wild-type group and lower in the JAK1 group except for the MSI-JAK1 vs. MSI-MHCI comparison in which positive enrichments are higher in the MSI-JAK1 group.
(XLSX)

## Abbreviations

CCLE: Cancer cell line encyclopedia
COAD: Colon adenocarcinoma
CRC: Colorectal carcinoma
EMT: Epithelial to mesenchymal transition
FWER: Family wise error rate
MSS: Microsatellite stable
MSI: Microsatellite instability
MSI-L/H: Microsatellite instability low/high
STAD: Stomach adenocarcinoma
TCGA: The cancer genome atlas
TPM: Transcripts per million
UCEC: Uterine corpus endometrial cancer
